# Down-regulation of lncRNA-NEF indicates poor prognosis in intrahepatic cholangiocarcinoma

**DOI:** 10.1042/BSR20181573

**Published:** 2019-05-21

**Authors:** Zhanqiang Liang, Bingshuai Zhu, Dongdong Meng, Xiwen Shen, Xuemin Li, Zhongzhen Wang, Liantao Li

**Affiliations:** Department of Hepatobiliary surgery, Zhengzhou Central Hospital Affiliated to Zhengzhou University, Zhengzhou City, Henan Province, 450007, P. R. China

**Keywords:** intrahepatic cholangiocarcinoma, lncRNA-NEF, metastasis, Runt-related transcription factor 1 (RUNX1), survival

## Abstract

LncRNA-NEF is a tumor suppressor lncRNA in liver cancer. The present study aimed to investigate the role of lncRNA-NEF in intrahepatic cholangiocarcinoma (IHCC), which is second most common type of primary cancer of the hepatobiliary system that causes high mortality rate. In the present study we found that lncRNA-NEF was down-regulated, while Runt-related transcription factor 1 (RUNX1) was up-regulated in tumor tissues than in adjacent healthy tissues of IHCC patients. Expression levels of lncRNA-NEF and RUNX1 were significantly and reversely correlated in tumor tissues but not in adjacent healthy tissues. Plasma levels of lncRNA-NEF were significantly lower in IHCC patients than in healthy controls. Down-regulation of lncRNA-NEF effectively distinguished stage I and II IHCC patients from healthy controls. Patients were followed up for 5 years, patients with high plasma levels of lncRNA-NEF showed significantly better survival conditions compared with patients with low expression levels of lncRNA-NEF. LncRNA-NEF overexpression led to inhibited expression of RUNX1 in cells of IHCC cell lines and inhibited cancer cell migration and invasion. In contrast, RUNX1 overexpression showed no significant effects on lncRNA-NEF expression, but attenuated the effects of lncRNA-NEF overexpression on cancer cell migration and invasion. We therefore concluded that lncRNA-NEF participated in IHCC possibly by interacting with RUNX1.

## Introduction

As a rare type of cholangiocarcinoma forms in the bile ducts, intrahepatic cholangiocarcinoma (IHCC) is heterogeneous hepatobiliary neoplasm with extremely poor prognosis [1]. IHCC only accounts for >8% of all hepatic cancer cases [2]. In spite of the low incidence rate, IHCC is still considered as a major cause of cancer-related deaths worldwide due to it highly aggressive nature [3]. Although a series of treatment strategies have been developed to treat IHCC, radical treatment strategies for this disease remain lack [4,5]. It is generally believed that the unclear pathogenesis is a major cause of poor treatment outcomes and high recurrence rate of IHCC [6]. Therefore, in-depth investigation on the pathogenesis of this disease may provide new insights to its treatment.

Runt-related transcription factor 1 (RUNX1), also refers to core-binding factor subunit α-2 (CBFA2) or acute myeloid leukemia 1 protein (AML1), is a transcription factor that regulates the downstream genes to participate in the differentiation of hematopoietic stem cells [7]. A growing body of literatures has shown that RUNX1 is a key player in cancer biology [8–10]. However, studies on the involvement of lncRNA in IHCC are rare. LncRNA-NEF is a tumor suppressor lncRNA that inhibits epithelial-to-mesenchymal transition (EMT) in liver cancer [11], while lncRNA-NEF promotes EMT during cancer development [12]. In the present study, we observed that lncRNA-NEF participated in IHCC possibly by interacting with RUNX1.

## Materials and methods

### Human specimens and cell lines

Our study enrolled 56 patients with IHCC and 42 healthy volunteer who were admitted by Zhengzhou Central Hospital from January 2010 to January 2013. Inclusion criteria of IHCC patients: (1) patients who were diagnosed by pathological biopsies; (2) patients completed treatment in Zhengzhou Central Hospital and follow-up study; (3) patients understood the experiment procedure and signed informed consent. Exclusion criteria: (1) patients with other diseases; (2) patients failed to complete treatment in Zhengzhou Central Hospital; (3) patients died of other causes during follow-up. The 42 healthy volunteers received systemic physiological examination in Zhengzhou Central Hospital and all of them showed normal physiological conditions. Healthy volunteers also signed informed consent. Patient group included 42 males and 14 females, and age ranged from 44 to 68 years, with a mean age of 52.1 ± 6.1 years. There were 12 cases in AJCC stage I, 12 in stage II, 14 in stage III and 28 in stage IV. Control group included 30 males and 12 females, and age ranged from 46 to 66 years, with a mean age of 51.4 ± 5.8 years. Two groups had similar age and gender distributions. Tumor tissue and adjacent healthy tissues specimens were obtained from each IHCC patients. Blood was extracted from both IHCC patients and healthy controls to prepare plasma. The research has been carried out in accordance with the World Medical Association Declaration of Helsinki.

HuCCT1 and TFK-1 human IHCC cell lines were purchased from Cell Bank of Australia. Cells of both cell lines were cultured with RPMI 1640 (Gibco, Carlsbad, CA, U.S.A.) containing 1% penicillin/streptomycin and 10% fetal bovine serum (FBS, Gibco, Carlsbad, CA, U.S.A.).

### Patient follow-up

All patients were follow-up for 5 years after discharge. Patients were visited every month until their deaths or the end of follow-up. Overall survival conditions were recorded, summarized and analyzed.

### Real-time quantitative PCR (RT-qPCR)

Total RNA was extracted from plasma, tumor tissues, healthy tissues and *in vitro* cultured cells using a Total RNA Purification Kit (Cat. 17200, Norgen Biotek). RNA concentration was measured using NanoDrop™ 2000 Spectrophotometers (Thermo Fisher Scientific, U.S.A.). Reverse transcription was performed using RevertAid RT Reverse Transcription Kit (Thermo Fisher Scientific). All PCR reaction systems were prepared using Luna® Universal One-Step RT-qPCR Kit (NEB). PCR reaction conditions were: 55 s at 95°C, and then 12 s at 95°C and 32 s at 58.5°C for 40 cycles. Sequences of primers used in PCR reactions were: 5′-CTGCCGTCTTAAACCAACCC-3′ (forward) and 5′-GCCCAAACAGCTCCTCAATT-3′ (reverse) for lncRNA-NEF; 5′-GGCAACTAACTGCTGGAACT-3′ (forward) and 5′-CTCATCTTGCCGGGGCTCAG-3′ (reverse) for RUNX1. 5′-GACCTCTATGCCAACACAGT3-′ (forward) and 5′- AGTACTTGCGCTCAGGAGGA-3′ (reverse) for β-actin. ABI PRISM 7500 qRT-PCR machine (Applied Biosystems, Rockford, IL, U.S.A.) was used to carry out all PCR reactions. 2^−ΔΔCT^ method was used to process all data.

### Cell transfection

Vectors expressing lncRNA-NEF and RUNX1 were designed and synthesized by GenePharma (Shanghai, China). Cells of HuCCT1 and TFK-1 were cultivated overnight to reach 70–80% confluence. Lipofectamine 2000 reagent (11668-019, Invitrogen, Carlsbad, U.S.A.) was used for all cell transfections with vectors at a dose of 15 mM. Cells without transfected were control cells. Cells transfected with empty vectors were negative control cells.

### Transwell migration and invasion assay

After transfected, cell migration and invasion were detected by Transwell migration and invasion assays only in cases of overexpression rate of both lncRNA-NEF and RUNX1 reached 200%. Briefly, cell suspensions (5 × 10^4^ cells/ml) were prepared using serum-free RPMI 1640 medium. Cells were transferred to the upper chamber with 0.1 ml cell suspension in each well, while the lower chamber was filled with RPMI 1640 medium containing 20% FBS. Cells were kept in an incubator (37°C, 5% CO_2_) for 24 h. Membranes were then collected and stained with 0.5% Crystal Violet (Sigma-Aldrich, U.S.A.) for 20 min at 25°C. Stained cells were counted under an optical microscope. Before invasion assay, the upper chamber was pre-coated with Matrigel (356234, Millipore, U.S.A.).

### Western blot

This experiment was performed in cases of overexpression rate of both lncRNA-NEF and RUNX1 reached 200%. Total protein was extracted from *in vitro* cultured cells using a Total Protein Extraction Kit (NBP2-37853, Novus Biologicals). After measurement of protein concentrations using Pierce BCA Protein Assay Kit (Thermo Fisher Scientific), protein samples were denatured and subjected to electrophoresis using 10% SDS-PAGE gel with 20 µg protein per lane. After blocking in serum-free milk at room temperature for 2 h, Western blot was performed using conventional method. Primary antibodies included rabbit anti-human RUNX1 (ab35962, 1:1400, Abcam) and GAPDH (ab9485, 1:1400, Abcam). The secondary antibody was goat-anti rabbit IgG-HRP secondary antibody (1:1000, MBS435036, MyBioSource). Pierce ECL Western Blotting Substrate (Thermo Fisher Scientific) was dropped onto the membranes to develop signals. Grey scale normalization was performed using ImageJ software.

### Statistical analysis

All experiments were performed in triplicate manner and data were recorded as mean ± standard deviation. Comparisons between two groups were performed by Student’s *t* test, and comparisons among multiple groups were performed by one-way analysis of variance followed by Tukey test. Diagnostic value of plasma lncRNA-NEF was analyzed by ROC curve with IHCC patients as true positive cases and healthy controls as true negative cases. Correlations between expression levels of lncRNA-NEF and RUNX1 mRNA were analyzed by Pearson Correlation Coefficient. Patients were divided into high expression group (*n* = 26) and low expression group (*n* = 30) according to the plasma levels of lncRNA-NEF using Youden index. Survival curves were plotted using Kaplan–Meier method and compared by log-rank test. *P*<0.05 was considered to be statistically significant.

## Results

### LncRNA-NEF and RUNX1 mRNA were differentially expressed in tumor tissues and adjacent healthy tissues of IHCC patients

**Figure 1:**
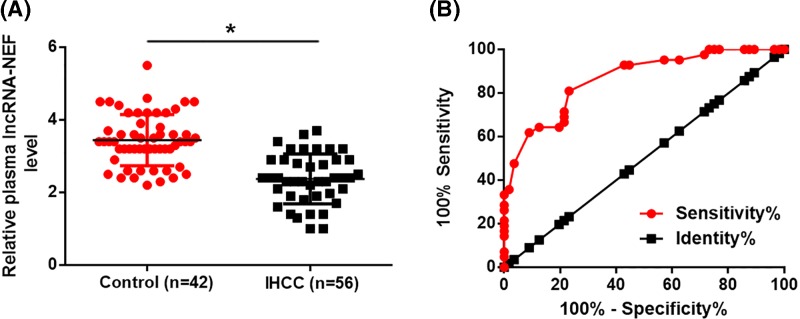
LncRNA-NEF and RUNX1 mRNA were differentially expressed in tumor tissues and adjacent healthy tissues of IHCC patients Compared with adjacent healthy tissues, expression levels of lncRNA-NEF were significantly increased (**A**), while expression levels of RUNX1 mRNA were significantly up-regulated (**B**) in tumor tissues of IHCC patients. *P<0.05.

Expression of lncRNA-NEF and RUNX1 mRNA in tumor tissues and adjacent healthy tissues of 56 patients with IHCC was detected by RT-qPCR. As showed in Figure 1A, compared with adjacent healthy tissues, expression levels of lncRNA-NEF were significantly decreased in tumor tissues of IHCC patients (*P*<0.05). In contrast, expression levels of RUNX1 mRNA were significantly up-regulated in tumor tissues than in healthy tissues (Figure 1B, *P*<0.05).

### Expression levels of lncRNA-NEF and RUNX1 were significantly and reversely correlated in tumor tissues but not in adjacent healthy tissues

**Figure 2:**
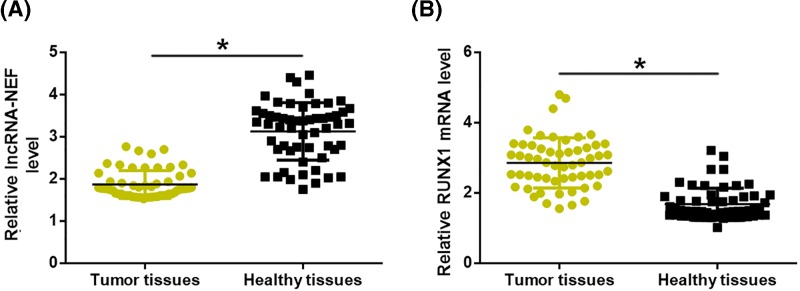
Expression levels of lncRNA-NEF and RUNX1 were significantly and reversely correlated in tumor tissues but not in adjacent healthy tissues. Pearson Correlation Coefficient revealed a significantly reverse correlation between the expression levels of lncRNA-NEF and RUNX1 mRNA in tumor tissues (**A**) but not in healthy tissues (**B**).

Correlations between expression levels of lncRNA-NEF and RUNX1 mRNA were analyzed by Pearson Correlation Coefficient. As shown in Figure 2A, a significantly reverse correlation was between the expression levels of lncRNA-NEF and RUNX1 mRNA in tumor tissues. In contrast, the correlation between expression levels of lncRNA-NEF and RUNX1 mRNA was not strong in healthy tissues (Figure 1B, *P*=0.6604).

### Down-regulation of lncRNA-NEF separated IHCC patients from healthy controls

**Figure 3:**
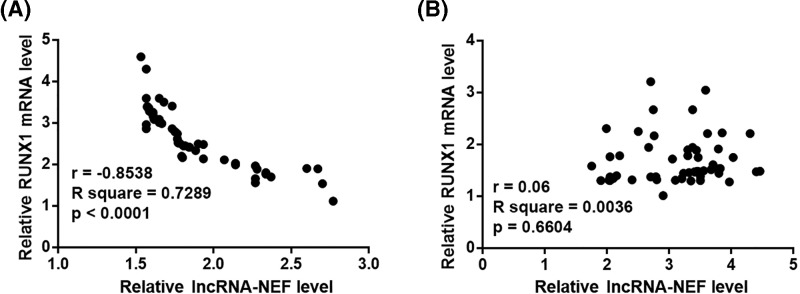
Down-regulation of lncRNA-NEF separated IHCC patients from healthy Down-regulation of lncRNA-NEF separated IHCC patients from healthy. Plasma levels of lncRNA-NEF were significantly lower in IHCC patients than in healthy controls (**A**) (*P<0.05). Down-regulation of lncRNA-NEF effectively distinguished stage I and II IHCC patients from healthy controls (**B**).

RT-qPCR results showed that, compared with control group, plasma levels of lncRNA-NEF were significantly decreased in IHCC patients (Figure 3A, *P*<0.05). Our study included 24 IHCC patients at stage I or II, which are the early stages of tumor development. Diagnostic value of plasma lncRNA-NEF for early stage IHCC was analyzed by ROC curve with IHCC patients as true positive cases and healthy controls as true negative cases. As shown in Figure 3B, area under the curve was 0.8642 (standard error: 0.03621; 95% confidence interval: 0.7932–0.9351; *P*<0.05).

### Low plasma levels of lncRNA-NEF indicated poor survival conditions in IHCC patients

**Figure 4:**
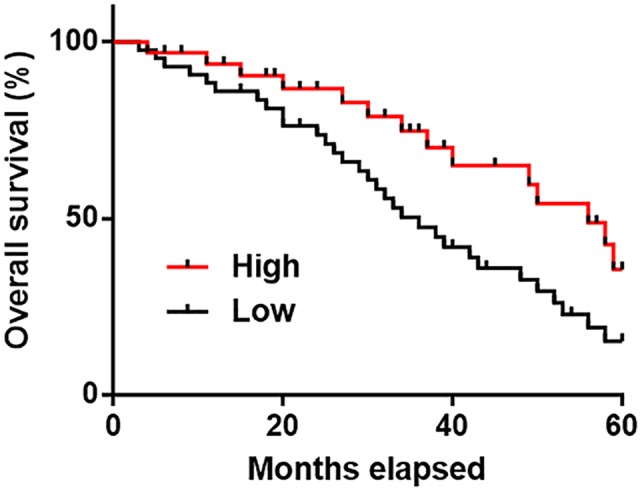
Low plasma levels of lncRNA-NEF indicated poor survival conditions in IHCC patients

Patients were divided into high expression group (*n*=26) and low expression group (*n*=30) according to the plasma levels of lncRNA-NEF using Youden index. Survival curves were plotted using Kaplan–Meier method and compared by log-rank test. As showed in Figure 4, patients with low plasma levels of lncRNA-NEF showed significantly worse overall survival compared with patients with high plasma levels of lncRNA-NEF (*P*=0.0198).

### LncRNA-NEF overexpression led to inhibited RUNX1 expression in IHCC cells

**Figure 5:**
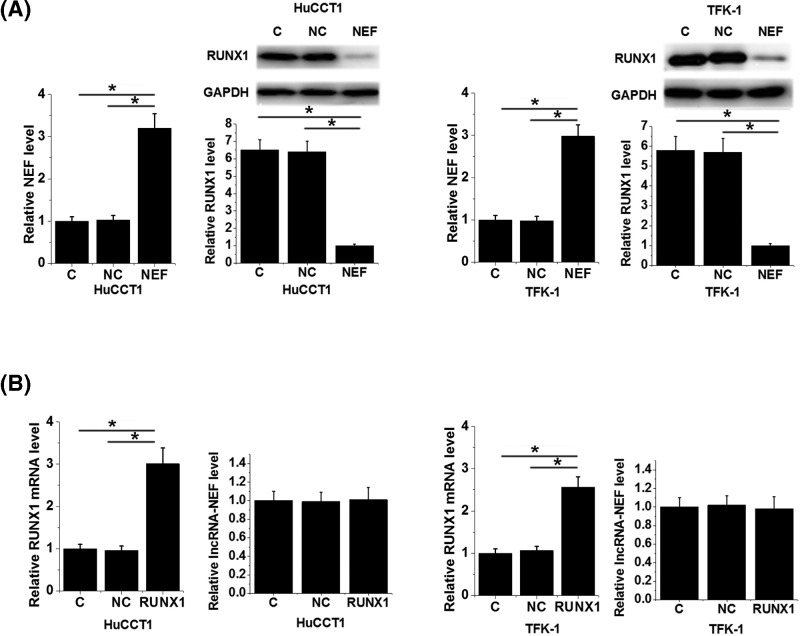
LncRNA-NEF overexpression led to inhibited RUNX1 expression in IHCC cells LncRNA-NEF overexpression led to significantly inhibited expression of RUNX1 in cells of both HuCCT1 and TFK-1 human IHCC cell lines (**A**). In contrast, RUNX1 overexpression did not significantly alter the expression of lncRNA-NEF in those cells (**B**) (*P<0.05).

Compared with control group (C) and negative control group (NC), lncRNA-NEF overexpression led to significantly inhibited expression of RUNX1 in cells of both HuCCT1 and TFK-1 human IHCC cell lines (Figure 5A). In contrast, RUNX1 overexpression did not significantly alter the expression of lncRNA-NEF in those cells (Figure 5B)

### LncRNA-NEF overexpression inhibited IHCC cell migration and invasion possibly through RUNX1

**Figure 6:**
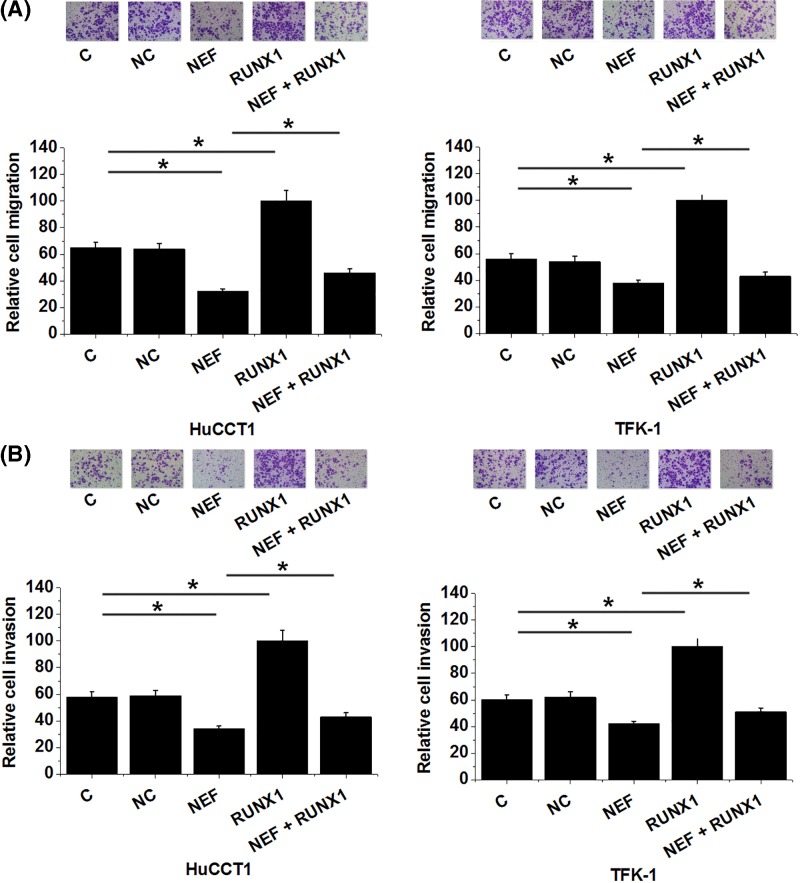
LncRNA-NEF overexpression inhibited IHCC cell migration and invasion possibly through RUNX1 LncRNA-NEF overexpression led to significantly inhibited, while RUNX1 led to significantly promoted migration (**A**) and invasion (**B**) of cells of both HuCCT1 and TFK-1 human IHCC cell lines. In addition, RUNX1 overexpression partially compensate the inhibitory effects of lncRNA-NEF overexpression on cancer cell migration and invasion (*P<0.05).

Compared with control group (C) and negative control group (NC), lncRNA-NEF overexpression led to significantly inhibited, while RUNX1 led to significantly promoted migration (Figure 6A) and invasion (Figure 6B) of cells of both HuCCT1 and TFK-1 human IHCC cell lines (*P*<0.05). In addition, RUNX1 overexpression partially compensates the inhibitory effects of lncRNA-NEF overexpression on cancer cell migration (Figure 6A) and invasion (Figure 6B) (*P*<0.05).

## Discussion

As a tumor suppressor lncRNA, lncRNA-NEF promotes EMT in liver cancer, while its involvement in other types of human malignancies remains unclear. In the present study, we showed that lncRNA-NEF is involved in the regulation of migration and invasion of cancer cells in IHCC patients. The actions of lncRNA-NEF in IHCC are likely achieved through the interactions with RUNX1, which is a well-characterized player in cancer biology.

Development of IHCC globally affects the expression of both coding and non-coding genes [13,14]. As a subgroup of non-coding RNAs composed of more than 200 nucleotides, lncRNAs are critical player in the progression of different types of human cancers including IHCC [15,16]. Down-regulation of lncRNA-NEF has been observed in liver cancer. In the present study, we observe that lncRNA-NEF was down-regulated in tumor tissues than in adjacent healthy tissues if IHCC patients. In addition, plasma lncRNA-NEF was up-regulated in ICHH patient than in healthy controls. Therefore, inhibited lncRNA-NEF expression may play a role in the pathology of IHCC.

At present, the treatment of IHCC is still challenged by the high prevalence of tumor metastasis [17]. Therefore, early diagnosis is still important for the survival of patients with IHCC. With the advantage of non-invasive nature, circulating biomarkers have been widely used in the diagnosis and prognosis of human cancers [18,19]. Our study enrolled 24 IHCC patients at stage I or II, which are the early stages of tumor development. ROC curve analysis showed that down-regulation of plasma circulating lncRNA-NEF can be used to effectively distinguish early stage IHCC patients from healthy controls, indicating the potentials of lncRNA-NEF in the early diagnosis of IHCC. In addition, patients with higher plasma levels of lncRNA-NEF showed much better overall survival compared with patients with lower plasma levels of lncRNA-NEF, indicating its values in the prediction of prognosis of IHCC.

RUNX1 is usually overexpressed in human cancers [20], and overexpression of RUNX1 is related to cancer progression and poor prognosis [21]. Consistent with previous studies, our study also observed significantly up-regulated RUNX1 in tumor tissues than in healthy tissues of IHCC patients. LncRNA-NEF and RUNX1 both participate in EMT, but their roles are opposite [11,12]. Based on the data, we have concluded that lncRNA-NEF is likely an upstream inhibitor of RUNX1 in IHCC cells. However, the regulatory effects of lncRNA-NEF on RUNX1 in IHCC is likely mediated by disease-related factors due to the factor that expression levels of lncRNA-NEF and RUNX1 were only positively correlated in tumor tissues but not in adjacent healthy tissues of IHCC patients.

In conclusion, lncRNA-NEF was down-regulated, while RUNX1 was up-regulated in IHCC. LncRNA-NEF may participate in IHCC by interacting with RUNX1.

## Abbreviations

AML1: acute myeloid leukemia 1 protein
EMT: epithelial-to-mesenchymal transition
IHCC: intrahepatic cholangiocarcinoma
RUNX1: Runt-related transcription factor 1
